# Impact of the preoperative prognostic nutritional index as a predictor for postoperative complications after resection of locally recurrent rectal cancer

**DOI:** 10.1186/s12885-021-08160-5

**Published:** 2021-04-20

**Authors:** Masakatsu Paku, Mamoru Uemura, Masatoshi Kitakaze, Shiki Fujino, Takayuki Ogino, Norikatsu Miyoshi, Hidekazu Takahashi, Hirofumi Yamamoto, Tsunekazu Mizushima, Yuichiro Doki, Hidetoshi Eguchi

**Affiliations:** Department of Gastroenterological Surgery; Graduated School of Medicine, Osaka University, 2-2 E2, Yamadaoka, Suita, Osaka, 565-0871 Japan

**Keywords:** Rectal cancer, Local recurrence, Postoperative complication, Inflammatory index, Nutrition

## Abstract

**Background:**

Local recurrence is common after curative resections for rectal cancer. Surgical intervention is among the best treatment choices. However, achieving a negative resection margin often requires extensive pelvic organ resections; thus, the postoperative complication rate is quite high. Recent studies have reported that the inflammatory index could predict postoperative complications. This study aimed to validate the correlation between clinical factors, including inflammatory markers, and severe complications after surgery for local recurrent rectal cancer.

**Methods:**

This retrospective study included 99 patients that underwent radical resections for local recurrences of rectal cancer. Postoperative complications were graded according to the Clavien-Dindo classification. Grades ≥3 were defined as severe complications. Risk factors for severe complications were identified with univariate and multivariate logistic regression models and assessed with receiver-operating characteristic curves.

**Results:**

Severe postoperative complications occurred in 38 patients (38.4%). Analyses of correlations between inflammatory markers and severe postoperative complications revealed that the strongest correlation was found between the prognostic nutrition index and severe postoperative complications. The receiver-operating characteristic analysis showed that the optimal prognostic nutrition index cut-off value was 42.2 (sensitivity: 0.790, specificity: 0.508). In univariate and multivariate analyses, a prognostic nutrition index ≤44.2 (Odds ratio: 3.007, 95%CI:1.171–8.255, *p* = 0.02) and a blood loss ≥2850 mL (Odds ratio: 2.545, 95%CI: 1.044–6.367, *p* = 0.04) were associated with a significantly higher incidence of severe postoperative complications.

**Conclusions:**

We found that a low preoperative prognostic nutrition index and excessive intraoperative blood loss were risk factors for severe complications after surgery for local recurrent rectal cancer.

**Supplementary Information:**

The online version contains supplementary material available at 10.1186/s12885-021-08160-5.

## Background

Colorectal cancer is one of the most common cancers in the world [1]. The 5-year survival rate for colorectal cancer has improved in the last 30 years, due to advancements in surgical and drug treatments. However, recurrences occur frequently after colorectal cancer surgery. In particular, the local recurrence of rectal cancer (LRRC) after a curative resection occurs at rates of 5.6 to 11% [2–4].

LRRC can be cured with radical resection. The reported 5-year survival rate is 43 to 70% after a curative resection for LRRC [5–7]. Local re-recurrences after LRRC surgery are often observed; therefore, a radical resection for LRRC often requires a highly invasive procedure, such as a total pelvic exenteration (TPE) combined with a sacral resection [8]. Consequently, postoperative complications occur frequently in LRRC at rates of 24 to 68% [9–12].

Recent studies have shown that inflammatory markers, such as the neutrophil/lymphocyte ratio (NLR) and the platelet/lymphocyte ratio (PLR), were useful for predicting postoperative complications and prognosis [13, 14]. These markers are calculated based on a complete blood count, which makes them simple and versatile. In addition, the prognostic nutritional index (PNI, based on serum albumin and lymphocyte counts) and the serum C-reactive protein (CRP)/serum albumin ratio (CAR) were shown to be effective predictors of postoperative complications after colorectal cancer surgery [15, 16]. However, no study has clarified the association between inflammatory markers and postoperative complications in LRRC.

The purpose of this study was to clarify the correlation between severe postoperative complications and various clinical factors, including inflammatory markers, in LRRC.

## Methods

### Study population

This retrospective cohort study was performed in Osaka University Hospital. We collected information for patients that had undergone curative resections for LRRC between January 2000 and January 2020. A total of 99 patients were eligible for this study.

### Patient and tumor characteristics

We acquired information on patient characteristics, including age at surgery, sex, body mass index (BMI), and the preoperative physical state, assessed with the American Society of Anesthesiologists (ASA) physical status classification. In addition, we examined the location and TNM stage of the primary tumor (based on the Union for International Cancer Control, 8th edition), the postoperative adjuvant therapy given after the initial treatment, and the preoperative treatment for LRRC surgery. We also included data on intraoperative details, including the surgical procedures, surgical approaches, combined resected organs, operation time, blood loss volume, and residual tumor (R status). We assessed the severity of postoperative complications with the Clavien-Dindo (CD) classification. Severe postoperative complications were defined as CD grade ≥ 3.

### Inflammatory markers

The latest laboratory data processed before the LRRC surgery was collected, including the complete blood count and the serum albumin and CRP levels. Serum CRP levels under 0.04 mg/L were below the detection limit of our measuring instrument; therefore, values under 0.04 mg/L were set to 0.04 mg/L for this analysis. These data were used to calculate the inflammatory markers, NLR, PLR, lymphocyte/CRP ratio (LCR), CAR, and PNI.

### Statistical analysis

All categorical data were presented as number of cases and percentages, while continuous data were shown as median and interquartile range (IQR). We performed logistic regression analyses to assess correlations between the incidence of severe postoperative complications and each inflammatory marker. We then created receiver operating characteristic (ROC) curves and compared areas under the ROC curves to determine the strongest effective marker of severe postoperative complications. We performed multiple logistic regression analyses to assess the correlation between severe postoperative complications and clinical factors.

All data were processed and analyzed with JMP Pro 14.0 (SAS Institute Inc., Cary NC). Two-tailed *p*-values < 0.05 were considered statistically significant. We calculated the exact 95% confidence intervals (95% CIs) for absolute differences and odds ratios (ORs).

This study was performed in accordance with the Declaration of Helsinki (1975, and revised in 2008). The study protocol was approved by the Ethics Committee of Osaka University Hospital. All study participants provided written informed consent.

## Results

### Clinical characteristics of the study population

Table 1 and Supplemental Table 1 show patient and primary tumor characteristics for the 99 patients. The cohort included 57 males and 42 females. The median age was 61 years [IQR 54.5–68]. The median BMI was 22.2 kg/m^2^ [IQR 20.2–24.1]. The majority of the patients were class 2 of preoperative physical state (*n* = 71; 71.7%). A total of 63 patients (63.6%) received neo-adjuvant therapy for LRRC. More than a half of the patients (*n* = 53; 53.5%) had chemoradiotherapy, whereas smaller numbers had chemotherapy (*n* = 9; 9.1%) or radiotherapy alone (*n* = 1; 1.0%). About primary tumor characteristics, more than 40% of the primary tumors were located in the lower rectum (*n* = 45; 45.6%). Regarding the T stage of the primary tumors, the majority of patients was T3 or T4 (T3, *n* = 48; 48.5%, T4, *n* = 35; 35.4%). About half of the patients had lymph node metastasis in the primary cancers (N1, *n* = 26; 26.3%, N2, *n* = 21; 21.2%). Adjuvant chemotherapy for the primary cancer was given to more than half of the patients (*n* = 56; 56.6%).

**Table 1:** Clinical characteristics of the patients with locally recurrent rectal cancer

Variable	Patients (***n*** = 99)
Patient characteristics	
Male sex, n (%)	57 (57.6%)
Age at surgery (years), median [IQR]	61 [54.5–68]
BMI (kg/m2), median [IQR]	22.2 [20.2–24.1]
ASA classification (1/2/3/unknown), n	15/71/5/8
Neo-adjuvant therapy	63 (63.6%)
Chemoradiotherapy, n (%)	53 (53.5%)
Chemotherapy alone, n (%)	9 (9.1%)
Radiotherapy alone, n (%)	1 (1.0%)

Values are the number of patients, unless indicated otherwise

*IQR* interquartile range, *BMI* body mass index, *ASA* American Society of Anesthesiologists

### Preoperative laboratory data and inflammatory makers of the study population

Table 2 shows the preoperative laboratory data results for the 99 patients. Regarding complete blood count, the median numbers of neutrophils, lymphocytes and platelets were within normal range. The median level of serum albumin (g/dL) was lower than normal value (value = 3.8 [IQR 3.4–4.1]). The median level of CRP was within normal range (value = 0.09 [IQR 0.04–0.46]. The inflammatory markers were calculated from the blood test data and each median value was as follow: NLR, 3.1, PLR, 232.5; CAR, 0.026; LCR, 11417.3; and PNI, 42.8.

**Table 2:** Preoperative laboratory data and inflammatory makers in 99 patients with locally recurrent rectal cancer

Variable	Value
CBC, median [IQR]	
Neutrophils (/μL)	3391.6 [2446.9–4440.0]
Lymphocytes (/μL)	1082.1 [764.6–1464.2]
Platelets (× 10^4^/μL)	24.1 [20.5–28.1]
Serum level, median [IQR]	
Albumin (g/dL)	3.8 [3.4–4.1]
CRP (mg/dL)	0.09 [0.04–0.46]
Inflammatory markers, median [IQR]	
Neutrophil/lymphocyte ratio	3.1 [2.3–4.4]
Platelet/lymphocyte ratio	232.5 [156.5–340.5]
lymphocyte/CRP ratio	11,417.3 [2526.4–22,872.9]
CRP/albumin ratio	0.026 [0.010–0.124]
Prognostic nutritional index	42.8 [38.4–46.8]

*CBC* complete blood count, *IQR* interquartile range, *CRP* C-reactive protein

### Operative details of the study population

Table 3 displays the intraoperative data for the 99 patients. The majority of the patients (*n* = 79; 79.8%) underwent a laparotomy and the rest of 20 patients (20.2%) underwent laparoscopic surgery. Regarding the type of surgery, the most common procedure was a tumorectomy or lateral lymph node dissection (*n* = 44; 44.4%) and a TPE was performed in 22 patients (22.2%). More than 40% of the patients (*n* = 40; 40.4%) underwent a combined sacral resection. The median operation time was 680 min [IQR 438–871], and the median intraoperative blood loss was 2850 mL [IQR 530–5240]. In histopathological outcome, the complete resection rate (R0) was 93.9% (*n* = 93).

**Table 3:** Intraoperative details for treating local recurrence of rectal cancer

Variable	Patients (***n*** = 99)
Approach, n (%)	
Laparotomy	79 (79.8%)
Laparoscopy	20 (20.2%)
Type of surgery, n (%)	
Tumorectomy / lateral lymph node dissection	44 (44.4%)
Low anterior resection	17 (17.2%)
Abdominoperineal resection	16 (16.2%)
Total pelvic exenteration	22 (22.2%)
Combined sacral resection, n (%)	40 (40.4%)
Operation time (minutes), median [IQR]	680 [438–871]
Blood loss (ml), median [IQR]	2850 [530–5240]
Residual tumor status, n (%)	
R0	93 (93.9%)
R1	6 (6.1%)

Values are the number of patients (%), unless indicated otherwise

*IQR* interquartile range

### Postoperative complications of the study population

The incidence of postoperative complications is shown in Table 4. Postoperative complications occurred in 79 patients (79.8%). Complications included a pelvic abscess in 36 patients (36.4%), perineal wound infections in 37 patients (37.4%), abdominal wound infections in 16 patients (16.2%), bleeding in 8 patients (8.1%), bowel obstruction or ileus in 20 patients (20.2%), urinary tract infections in 21 patients (21.2%), and venous thromboembolism (VTE) in 5 patients (5.1%). The postoperative complications were classified as follows, 41 patients (41.4%) had CD grade I or II, 36 patients (36.4%) had CD grade III or IV, and 2 patients (2.0%) had CD grade V.

**Table 4:** Postoperative complications in patients treated for local recurrence of rectal cancer

Complication	Patients (***n*** = 99)
No complications, n (%)	20 (20.2%)
Total complications, n (%)	79 (79.8%)
Perineal wound infection	37 (37.4%)
Pelvic abscess	36 (36.4%)
Urinary tract infection	21 (21.2%)
Bowel obstruction / ileus	20 (20.2%)
Abdominal wound infection	16 (16.2%)
Bleeding	8 (8.1%)
Venous thromboembolism	5 (5.1%)
Others	44 (44.4%)
Clavien-Dindo classification, n (%)	
Grade I or II	41 (41.4%)
Grade III or IV	36 (36.4%)
Grade V	2 (2.0%)

### Correlation between inflammatory markers and severe postoperative complications

**Fig. 1:**
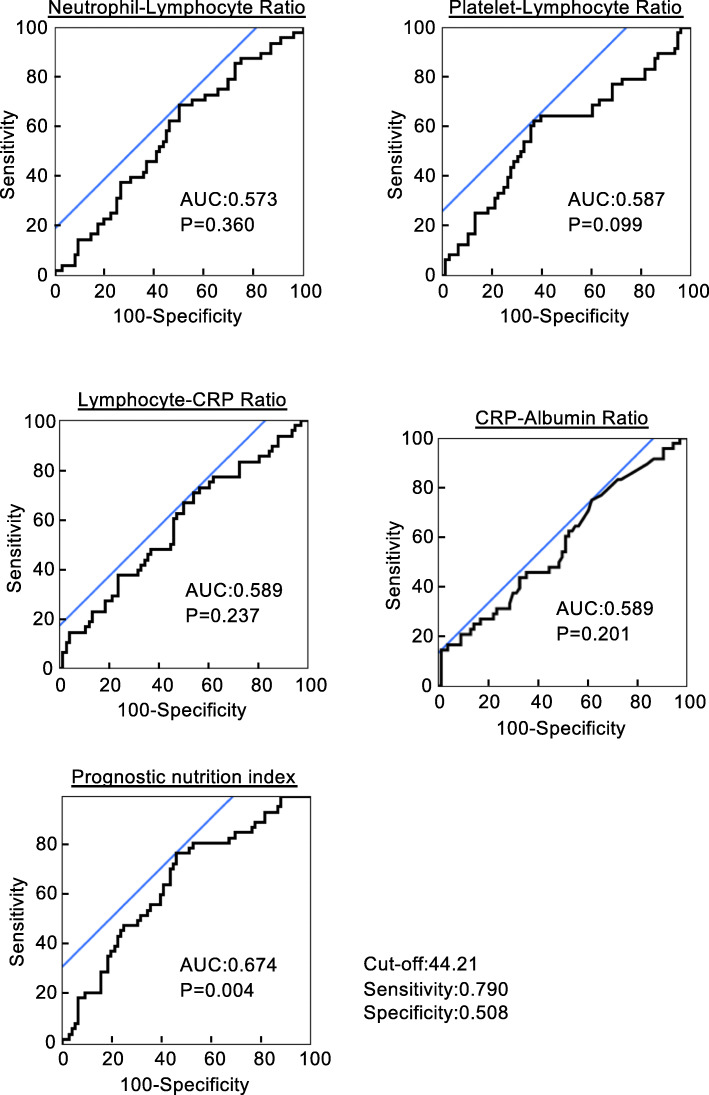
Receiver operating characteristic curves analysis to evaluate the predictive value of each inflammatory marker for postoperative complications in patients with local recurrent rectal cancer (LRRC). Preoperative prognostic nutrition index showed the highest accuracy for the prediction of postoperative complications compared with neutrophil-lymphocyte ratio (NLR), platelet-lymphocyte ratio (PLR), lymphocyte-CRP ratio (LCR) and CRP-albumin ratio (CAR) in patients with LRRC

To identify correlations between inflammatory markers and severe postoperative complications in LRRC, we performed univariate analyses. Then, we created ROC curves to analyze the sensitivity and specificity of each inflammatory marker that was correlated to severe postoperative complications (Fig. 1). The area under the curve (AUC) and *p*-value for each inflammatory marker are shown in each panel. The PNI showed the strongest correlation with severe postoperative complications in LRRC. The PNI cutoff value for indicating severe complications was 44.2, and it showed a sensitivity of 0.790 and a specificity of 0.508.

### Risk factors for severe postoperative complications

Potential risk factors for severe postoperative complications in LRRC were analyzed with univariate and multivariate analyses based on logistic regression. As shown in Table 5, the univariate analysis showed that the PNI and intraoperative blood loss were significantly correlated with severe postoperative complications. The multivariate analysis of these factors revealed that a PNI < 44.2 (OR: 3.007, 95%CI: 1.171–8.255, *p* = 0.02) and blood loss ≥2850 mL (OR: 2.545, 95%CI: 1.044–6.367, *p* = 0.04) were independent risk factors (Table 6).

**Table 5:** Univariate analysis of clinical characteristics associated with severe postoperative complications (CD ≥ III)

Factors	Category	No. of patients	Incidence of severe complications (%)	OR (95% CI)	*p* value
Sex	Male	57	31.6%	0.105 (0.220–1.153)	0.105
	Female	42	47.6%		
Age, y	< 61	47	40.4%	1.179 (0.523–2.665)	0.691
	≥ 61	52	36.5%		
BMI, kg/m^2^	< 22.2	47	44.7%	1.663 (0.738–3.800)	0.583
	≥ 22.2	52	32.7%		
PNI	< 44.2	60	50.0%	3.875 (1.586–10.311)	0.003
	≥ 44.2	39	20.5%		
Surgical approach	Laparotomy	79	41.8%	2.152 (0.750–7.146)	0.159
	Laparoscopic	20	25.0%		
Operative time, min	< 680	49	30.6%	0.518 (0.224–1.171)	0.114
	≥ 680	50	46.0%		
Blood loss, ml	< 2850	49	24.5%	0.299 (0.124–0.692)	0.005
	≥ 2850	50	52.0%		
Sacrectomy	Yes	40	50.0%	2.278 (0.997–5.296)	0.051
	No	59	30.5%		

*CD* Clavien-Dindo classification, *OR* odds ratio, *CI* confidence interval, *BMI* body mass index, *PNI* prognostic nutritional index

**Table 6:** Multivariate analysis of clinical characteristics associated with severe postoperative complications (CD ≥ III)

Factors	OR (95% CI)	*p* value
PNI < 44.2	3.007 (1.171–8.255)	0.022
Blood loss ≥2850 mL	2.545 (1.044–6.367)	0.040

*CD* Clavien-Dindo classification, *OR* odds ratio, *CI* confidence interval, *PNI* prognostic nutritional index

## Discussion

Postoperative complications are known to be associated with a poor prognosis in colorectal cancer [17]. We found a postoperative complication rate of 79.8% in LRRC, and the rate of CD ≥ III complications was 38.4%. To avoid postoperative complications, it is important to know the risk factors and provide a presurgical therapeutic intervention to prevent them. We identified two independent risk factors for complications after LRRC surgery in this study: a preoperative PNI < 44.2 and intraoperative blood loss ≥2850 mL. These findings indicated that the preoperative nutritional status and surgical invasiveness could be related to severe postoperative complications in patients with LRRC.

The PNI is a simple nutritional index, based on the serum albumin level and the lymphocyte count. It has been associated with perioperative complications in various carcinomas [18, 19]. Albumin is an index of nutritional capacity, and lymphocytes are an index of nutrition and immune capacity. Thus, the PNI reflects nutrition and immune status. Accordingly, patients with low PNIs are expected to have low wound healing ability and low immune function.

One might ask why did severe complications after LRRC surgery show a stronger correlation with PNI than with other inflammatory markers? One explanation might be that the NLR and PLR values after chemotherapy did not reflect the nutrition and immune status correctly. Chemotherapy reduces blood cell counts by suppressing bone marrow activity; therefore, the NLR and PLR might be affected, because they are calculated from blood cell counts. In the present study, preoperative chemotherapy for LRRC was performed in 62 patients (62.6%). As a result, the NLR and PLR might be less sensitive to severe postoperative complications than expected. Another potential explanation might be the limited accuracy of our blood test equipment. Because we could not assess CRP levels below 0.04, CRP levels less than 0.04 were treated as 0.04 in this study. Therefore, we could not accurately assess the CAR and LCR values, which required the CRP level.

The relationship between blood loss and postoperative complications was previously reported in colorectal cancer surgery [20, 21]. Heavy bleeding can change the hemodynamics and impair organs, particularly the kidneys and liver, and it also affects coagulation. These changes can lead to several postoperative complications, such as VTE and bleeding. It has been reported that VTE and bleeding at or greater than CD grade III after curative resection of primary colorectal cancer occurs at rates of 0 to 0.16% and 0.2 to 0.81%, respectively [22–24]. Compared with these data, in the present study, VTE and bleeding events more frequently occurred (Table 4).

Some previous studies have reported that preoperative nutritional interventions were effective for postoperative outcomes in various types of cancer. Indeed, preoperative exercise and nutritional support improved the postoperative outcome in patients with gastric cancer [25]. Despite concern that nutritional interventions might delay surgery, preoperative treatments are often performed in patients with LRRC; therefore, there should be sufficient time to improve the patient’s nutritional status.

We previously reported that laparoscopic surgery was safe and useful for LRRC [26]. The magnifying effect of the laparoscope is highly effective in surgery for pelvic organ cancers, where it is difficult to expand the field of view. Additionally, the carbon dioxide insufflation used to create a working space in the abdomen can provide pressure, which reduces bleeding. Indeed, patients with rectal cancer that underwent laparoscopic surgery had less intraoperative blood loss than patients that underwent a laparotomy [27, 28]. Although laparoscopic surgery for LRRC could potentially prevent intraoperative and postoperative complications, the number of patients that underwent laparoscopic surgery in this study was insufficient to assess the correlation between the surgical approach and complications.

This study had some limitations. First, the study was retrospective, and data were from a single center. Second, the cohort was relatively small. Although 99 patients represented a relatively large cohort in clinical research for LRRC, it was insufficient to clarify the risk factors for postoperative complications.

## Conclusions

In conclusion, we found that a PNI < 44.2 and blood loss ≥2850 mL were independent risk factors for severe complications after LRRC surgery. Preoperative interventions that improve the nutritional status and an approach that reduces surgical invasiveness could potentially reduce the risk of severe complications after LRRC surgery.

## Supplementary Information

**Additional file 1: Supplemental Table 1.** Primary tumor characteristics of the patients in this study.

## Data Availability

The datasets used and/or analysed during the current study available from the corresponding author on reasonable request.

## Abbreviations

LRRC: Local recurrence of rectal cancer
TPE: Total pelvic exenteration
NLR: Neutrophil/lymphocyte ratio
PLR: Platelet/lymphocyte ratio
PNI: Prognostic nutritional index
CRP: C-reactive protein
CAR: CRP/serum albumin ratio
BMI: Body mass index
ASA: American Society of Anesthesiologists
CD: Clavien-Dindo
LCR: Lymphocyte/CRP ratio
IQR: Interquartile range
OR: Odds ratio
95% CI: 95% confidence interval
AUC: Area under curve

## References

[1] Ferlay J, Soerjomataram I, Dikshit R, Eser S, Mathers C, Rebelo M, Parkin DM, Forman D, Bray F (2015). Cancer incidence and mortality worldwide: sources, methods and major patterns in GLOBOCAN 2012. Int J Cancer.

[2] Peeters KC, Marijnen CA, Nagtegaal ID, Kranenbarg EK, Putter H, Wiggers T, Rutten H, Pahlman L, Glimelius B, Leer JW, van de Velde C, Dutch Colorectal Cancer Group (2007). The TME trial after a median follow-up of 6 years: increased local control but no survival benefit in irradiated patients with resectable rectal carcinoma. Ann Surg.

[3] van Gijn W, Marijnen CA, Nagtegaal ID, Kranenbarg EM, Putter H, Wiggers T, Rutten HJ, Påhlman L, Glimelius B, van de Velde CJ (2011). Preoperative radiotherapy combined with total mesorectal excision for resectable rectal cancer: 12-year follow-up of the multicentre, randomised controlled TME trial. Lancet Oncol.

[4] Beppu N, Kimura F, Aihara T, Doi H, Tomita N, Yanagi H, Yamanaka N (2017). Patterns of local recurrence and oncologic outcomes in T3 low rectal cancer (≤5 cm from the anal verge) treated with short-course radiotherapy with delayed surgery : outcomes in T3 low rectal cancer treated with short-course radiotherapy with delayed surgery. Ann Surg Oncol.

[5] Pacelli F, Tortorelli AP, Rosa F, Bossola M, Sanchez AM, Papa V, Valentini V, Doglietto GB (2010). Locally recurrent rectal cancer: prognostic factors and long-term outcomes of multimodal therapy. Ann Surg Oncol.

[6] Kanemitsu Y, Hirai T, Komori K, Kato T (2010). Prediction of residual disease or distant metastasis after resection of locally recurrent rectal cancer. Dis Colon Rectum.

[7] Harris CA, Solomon MJ, Heriot AG, Sagar PM, Tekkis PP, Dixon L, Pascoe R, Dobbs BR, Frampton CM, Harji DP, Kontovounisios C, Austin KK, Koh CE, Lee PJ, Lynch AC, Warrier SK, Frizelle FA (2016). The outcomes and patterns of treatment failure after surgery for locally recurrent rectal Cancer. Ann Surg.

[8] Uemura M, Ikeda M, Yamamoto H, Kitani K, Tokuoka M, Matsuda K, Hata Y, Mizushima T, Takemasa I, Sekimoto M, Hosokawa K, Matsuura N, Doki Y, Mori M (2011). Clinicopathological assessment of locally recurrent rectal cancer and relation to local re-recurrence. Ann Surg Oncol.

[9] Dresen RC, Gosens MJ, Martijn H, Nieuwenhuijzen GA, Creemers GJ, Daniels-Gooszen AW, van den Brule AJ, van den Berg HA, Rutten HJ (2008). Radical resection after IORT-containing multimodality treatment is the most important determinant for outcome in patients treated for locally recurrent rectal cancer. Ann Surg Oncol.

[10] Asoglu O, Karanlik H, Muslumanoglu M, Igci A, Emek E, Ozmen V, Kecer M, Parlak M, Kapran Y (2007). Prognostic and predictive factors after surgical treatment for locally recurrent rectal cancer: a single institute experience. Eur J Surg Oncol.

[11] Palmer G, Martling A, Cedermark B, Holm T (2007). A population-based study on the management and outcome in patients with locally recurrent rectal cancer. Ann Surg Oncol.

[12] Uemura M, Ikeda M, Sekimoto M, Haraguchi N, Mizushima T, Yamamoto H, Takemasa I, Ishii H, Mori M (2009). Prevention of severe pelvic abscess formation following extended radical surgery for locally recurrent rectal cancer. Ann Surg Oncol.

[13] Templeton AJ, Ace O, McNamara MG, Al-Mubarak M, Vera-Badillo FE, Hermanns T, Seruga B, Ocaña A, Tannock IF, Amir E (2014). Prognostic role of platelet to lymphocyte ratio in solid tumors: a systematic review and meta-analysis. Cancer Epidemiol Biomark Prev.

[14] Choi WJ, Cleghorn MC, Jiang H, Jackson TD, Okrainec A, Quereshy FA (2015). Preoperative neutrophil-to-lymphocyte ratio is a better prognostic serum biomarker than platelet-to-lymphocyte ratio in patients undergoing resection for nonmetastatic colorectal cancer. Ann Surg Oncol.

[15] Shibutani M, Maeda K, Nagahara H, Iseki Y, Ikeya T, Hirakawa K (2016). Prognostic significance of the preoperative ratio of C-reactive protein to albumin in patients with colorectal cancer. Anticancer Res.

[16] Mohri Y, Inoue Y, Tanaka K, Hiro J, Uchida K, Kusunoki M (2013). Prognostic nutritional index predicts postoperative outcome in colorectal cancer. World J Surg.

[17] Aoyama T, Oba K, Honda M, Sadahiro S, Hamada C, Mayanagi S, Kanda M, Maeda H, Kashiwabara K, Sakamoto J, Saji S, Yoshikawa T (2017). Impact of postoperative complications on the colorectal cancer survival and recurrence: analyses of pooled individual patients' data from three large phase III randomized trials. Cancer Med.

[18] Okada S, Shimada J, Teramukai S, Kato D, Tsunezuka H, Miyata N, Ishihara S, Furuya T, Nakazono C, Ishikawa N, Inoue M (2018). Risk stratification according to the prognostic nutritional index for predicting postoperative complications after lung cancer surgery. Ann Surg Oncol.

[19] Kanda M, Fujii T, Kodera Y, Nagai S, Takeda S, Nakao A (2011). Nutritional predictors of postoperative outcome in pancreatic cancer. Br J Surg.

[20] Egenvall M, Mörner M, Påhlman L, Gunnarsson U (2014). Degree of blood loss during surgery for rectal cancer: a population-based epidemiologic study of surgical complications and survival. Color Dis.

[21] Mörner ME, Gunnarsson U, Jestin P, Svanfeldt M (2012). The importance of blood loss during colon cancer surgery for long-term survival: an epidemiological study based on a population based register. Ann Surg.

[22] Pak J, Ikeda M, Uemura M, Miyake M, Nishikawa K, Miyamoto A, Miyazaki M, Hirao M, Nakamori S, Sekimoto M (2018). Risk factors for bleeding in patients receiving fondaparinux after colorectal cancer surgery. J Anus Rectum Colon.

[23] Yamaoka Y, Ikeda M, Ikenaga M, Haraguchi N, Miyake M, Sekimoto M (2015). Safety and efficacy of fondaparinux for prophylaxis of venous thromboembolism after colorectal cancer resection: a propensity score matched analysis. Dig Surg.

[24] Hata K, Kimura T, Tsuzuki S, Ishii G, Kido M, Yamamoto T, Sasaki H, Miki J, Yamada H, Furuta A, Miki K, Egawa S (2016). Safety of fondaparinux for prevention of postoperative venous thromboembolism in urological malignancy: a prospective randomized clinical trial. Int J Urol.

[25] Yamamoto K, Nagatsuma Y, Fukuda Y, Hirao M, Nishikawa K, Miyamoto A, Ikeda M, Nakamori S, Sekimoto M, Fujitani K, Tsujinaka T (2017). Effectiveness of a preoperative exercise and nutritional support program for elderly sarcopenic patients with gastric cancer. Gastric Cancer.

[26] Uemura M, Ikeda M, Kawai K, Nishimura J, Takemasa I, Mizushima T, Yamamoto H, Sekimoto M, Doki Y, Mori M (2018). Laparoscopic surgery using a Gigli wire saw for locally recurrent rectal cancer with concomitant intraperitoneal sacrectomy. Asian J Endosc Surg.

[27] Kennedy RH, Francis EA, Wharton R, Blazeby JM, Quirke P, West NP, Dutton SJ (2014). Multicenter randomized controlled trial of conventional versus laparoscopic surgery for colorectal cancer within an enhanced recovery programme: EnROL. J Clin Oncol.

[28] van der Pas MH, Haglind E, Cuesta MA, Fürst A, Lacy AM, Hop WC, Bonjer HJ (2013). Laparoscopic versus open surgery for rectal cancer (COLOR II): short-term outcomes of a randomised, phase 3 trial. Lancet Oncol.

