# Supplementary material for: Impact of the preoperative prognostic nutritional index as a predictor for postoperative complications after resection of locally recurrent rectal cancer
Source: BMC Cancer. 2021 Apr 20;21:435. doi: 10.1186/s12885-021-08160-5 (PMC8056720; doi:10.1186/s12885-021-08160-5)
Supplement: Supplementary file 1 — Additional file 1: Supplemental Table 1. Primary tumor characteristics of the patients in this study. [file 12885_2021_8160_MOESM1_ESM.docx]

| **Supplemental Table 1** Primary tumor characteristics of the patients in this study | |
| --- | --- |
| **Variable** | **Patients (n = 99)** |
| Primary tumor characteristics |  |
| Location (Upper rectum/Lower rectum/unknown), n | 45/45/9 |
| T stage (is/1/2/3/4/unknown), n | 1/2/10/48/35/3 |
| N stage (0/1/2/unknown) | 48/26/21/4 |
| M stage (0/1/unknown) | 82/7/10 |
| Adjuvant chemotherapy, n (%) | 56 (56.6%) |
| Values are the number of patients, unless indicated otherwise. | |
